# Anomalous Network Traffic Detection Method Based on an Elevated Harris Hawks Optimization Method and Gated Recurrent Unit Classifier

**DOI:** 10.3390/s22197548

**Published:** 2022-10-05

**Authors:** Yao Xiao, Chunying Kang, Hongchen Yu, Tao Fan, Haofang Zhang

**Affiliations:** School of Data Science and Technology, Heilongjiang University, Harbin 150000, China

**Keywords:** Gated recurrent unit, Harris Hawks optimization, feature selection, deep learning

## Abstract

In recent years, network traffic contains a lot of feature information. If there are too many redundant features, the computational cost of the algorithm will be greatly increased. This paper proposes an anomalous network traffic detection method based on Elevated Harris Hawks optimization. This method is easier to identify redundant features in anomalous network traffic, reduces computational overhead, and improves the performance of anomalous traffic detection methods. By enhancing the random jump distance function, escape energy function, and designing a unique fitness function, there is a unique anomalous traffic detection method built using the algorithm and the neural network for anomalous traffic detection. This method is tested on three public network traffic datasets, namely the UNSW-NB15, NSL-KDD, and CICIDS2018. The experimental results show that the proposed method does not only significantly reduce the number of features in the dataset and computational overhead, but also gives better indicators for every test.

## 1. Introduction

The network environment has gotten more complex in recent years as the internet advances. While people use the Internet’s convenience, several types of cyber-attacks come with it. This is still needed to be addressed to network security issues. This issue affects the performance of anomalous network traffic detection systems. Machine Learning has been introduced to detect anomalous network traffic detection systems because of the proliferation of network traffic. Moreover, network traffic includes a large number of feature information and carries various redundant features. Redundant features are features that can be difficult to detect and include features that are highly correlated with the rest of the detection targets and also features that have low correlations with the detection target. If there are too many redundant features in the network traffic data, it will add time complexity and space complexity to the algorithm, reduce the detection capability of the anomalous network traffic detection system and increase the computational overhead of the algorithm. It is a very difficult field for anomalous network traffic detection to select the best subset of features from the original data that benefit from the detection methods.

## 2. Related Works

Because of its high computational overhead and its low search efficiency, the random search algorithm used in traditional feature selection is difficult to meet the demand for real-time performance in anomalous traffic detection systems. Because of their faster computational speed, meta-heuristic search algorithms are becoming another alternative to feature selection algorithms by increasing detection accuracy and efficiency [1]. The current research literature is focused on Ant Colony Optimization (ACO), particle swarm optimization (PSO), Whale Optimization Algorithm (WOA), Genetic Algorithm (GA), etc. In [2], the output of the classifier is input into a different Support Vector Machine (SVM) to train the final detection model and an integrated intrusion detection model is obtained. However, it is difficult for algorithms like to support vector machines to respond quickly to intrusive behaviors. Song et al. [3] combines the XGBoost algorithm with the improved particle swarm optimization algorithm for parameter optimization in an easy solution to the continuous multivariable optimization problem. The work of Junior et al. [4] uses PSO to XGBoost to classify new coronary pneumonia images and improve the accuracy rate. However, when the volume of data is large, the complexity of XGBoost is high, and the overhead in space and time is relatively large. Mehdi et al. [5] proposed a method for solving feature subsets by ACO, describing how the ACO algorithm is solving feature subsets, but the ACO algorithm has the disadvantage that it is very easy to fall into local optimum.Mehrnaz et al. [6] proposed an intrusion detection model based on the Artificial Bee Colony algorithm (ABC) and AdaBoost. The model uses ABC in the selection of features, and then uses AdaBoost for classification. However, this method does not improve ABC, and it is easy to fall into local optimum and unstable optimum and unstable. Zhang et al. [7] proposed a Genetic Algorithm (GA) with a Deep Boltzmann Net (DBN) model for intrusion detection, which not only gives high accuracy for common types of attacks, but also for small training sets like U2R, but the use of a single dataset does not achieve overall detection accuracy. In the following work, some algorithms in related works will be selected for comparison.

Based on the above research, this paper proposes an approach to anomalous traffic detection using EHHO-GRU. This method is the first to use the relatively novel Harris Hawks Optimisation (HHO) algorithm as a feature selector that extracts a wide range of features from anomalous network traffic data and designs a unique fitness function to optimize the convergence speed of the overall abnormal traffic detection method. The HHO feature selector is compared to the optimized Elevated Harris Hawks Optimisation-Gated Recurrent Unit(EHHO) feature selector proposed in this paper. For the first time, the EHHO feature selector is combined with a GRU neural network to train and test the feature subset data solved from multiple common datasets. The dimensionality of the feature is significantly reduced, the computational overhead is reduced and the detection index of the neural network for abnormal traffic is improved.

## 3. Materials and Methods

### 3.1. GRU Neural Network

Recurrent Neural Network (RNN) [8] is a deep learning network for processing serialized data. By introducing recurrent connections between hidden layer units in adjacent time steps, RNN can effectively use historical information and it can make decisions on time series data, so it is widely used in the field of network traffic detection [9,10,11]. However, the phenomenon of exploding or vanishing gradients after multi-stage propagation of all time series data will cause the neural network to lose its long-term learning ability. This phenomenon is called exploding gradients and vanishing gradients.

Therefore, Long-Short Term Memory Neural Network (LSTM) [12] was proposed to solve the long-term propagation gradient problem. The long short-term memory neural network not only retains the recurrent memory capability of the outer hidden layer unit of the RNN, but also has three special gating systems inside its unit. Including forget gate, input gate, and output gate. It can control the length and weight of memory retention, so it is also introduced into the field of abnormal traffic detection [13]. However, the algorithmic complexity of LSTM with three more gating system parameters also increases greatly, resulting in extremely expensive training of LSTM.

**Figure 1:**
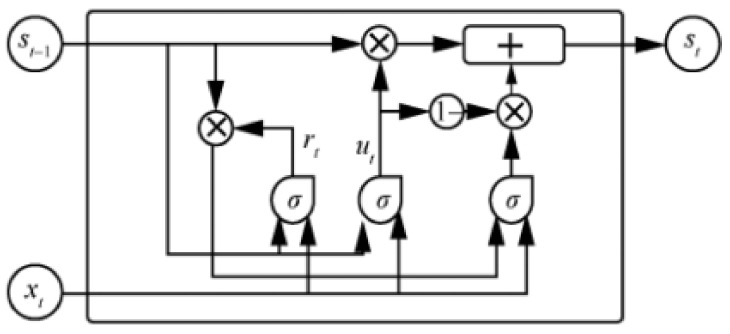
Gated Recurrent hidden layer unit.

To solve the RNN gradient problem and the high training cost of LSTM at the same time, Gated Recurrent Unit (GRU) was proposed by Cho et al. [14] Compared to LSTM with three gating systems, GRU has only two gating systems: an update gate and a reset gate. In this paper, a gated recurrent unit is used as a detector for abnormal flow. The structure of the GRU hidden layer unit is shown in Figure 1, where $S_{t}$ denotes the state of the hidden layer unit at time step *t*, which is jointly determined by the hidden layer unit state $S_{t-1}$ at the previous moment and the input $X_{t}$ at the current time step *t*, where rt is the reset gate at time step *t* and $u_{t}$ is the update gate at time step *t*. The hidden layer unit state $S_{t}$ at time step *t* is also used as the input for the next time step.

In the GRU hidden layer unit of Figure 1, the output of the current time step is given by the following equation:

(1) $$S_{t}=\left(1-u_{t}\right)⊙h+u_{t}⊙S_{t-1}$$

where *h* denotes the candidate hidden layer state at time step *t*, ⊙ means that two variables are multiplied according to their corresponding elements, and its equation is:

(2) $$h=\tanh(U^{h}X_{t}+W^{h}\left(S_{t-1}⊙r_{t}\right))$$

The update gate $u_{t}$ and the reset gate $r_{t}$ can ignore the candidate hidden layer state $c_{t}$ and the previous hidden layer state $S_{t–1}$ independently of each other. The hidden layer state $S_{t-1}$ and the input $X_{t}$ jointly control the update gate $u_{t}$ and the reset gate $r_{t}$, and output a value in [0,1] after compression by the activation function sigmoid, which is used to denote the degree of activation for update gate $u_{t}$ and reset gate $r_{t}$. In short, if the activation function output is 0, the update gate will keep all candidate hidden layer states *h*, and the reset gate will ignore all hidden layer states $S_{t-1}$ of the previous time step. The equations for updating the gate $u_{t}$ and resetting the gate $r_{t}$ are:

(3) $$u_{t}=\sigma \left(U^{u}X_{t}+W^{u}S_{t-1}\right)$$

(4) $$r_{t}=\sigma \left(U^{r}X_{t}+W^{r}S_{t-1}\right)$$

$U^{u}$, $U^{r}$, $U^{h}$ denotes the weight matrix from the input unit to the update gate, reset gate, and hidden layer unit respectively, $W^{u}$, $W^{r}$, $W^{h}$ denotes the weight matrix from the hidden layer unit to the update gate, reset gate and hidden layer unit respectively. And σ means the sigmoid function.

### 3.2. Harris Hawks Optimization

Harris Hawks Optimization (HHO) is a meta-heuristic algorithm proposed by Heidari et al. [15] in 2019. It was inspired by the predation behavior of the Harris hawk on its prey (hares). The algorithm outperformed other well-known algorithms, including PSO, GA, GOA, ALO, WOA, BOA, and SMA. Further, the algorithm was tested on 29 benchmark problems and other tasks that represent real-world engineering tasks [15]. The experiments had shown very competitive results The algorithm has attracted much attention since its inception and has been applied to many fields. Chen Huiling et al. [16] used the HHO to identify the parameters of photovoltaic cells and modules. The research shows that the new algorithm has good optimization performance, but no one has applied it to the intrusion detection system for feature selection of abnormal flow data.

HHO uses mathematical equations to simulate the prey mechanism of Harris hawks in different situations. In the Harris Hawks Optimization, the prey gradually approaches the optimal solution with the number of iterations, and the candidate solution is the Harris Hawk. The overall algorithm includes two parts: the exploration and exploitation phases. Exploration versus exploitation: exploring unknown actions to gain more information versus exploiting the information already collected.

#### 3.2.1. Exploration Phase

In this phase, all Harris hawks are considered candidate solutions. In each iteration, the fitness value is computed for all these possible solutions based on the intended prey. Exploitation refers to a local search around the area acquired during the exploration phase. In the exploration phase, Harris Hawks first waits, after which it monitors and evaluates the search space [*lb*, *ub*]. And randomly search for prey under two strategies, update the position with q as the probability during iteration, and its mathematical expression is:

(5) $$X_{t+1}=\left\{\begin{array}{c} X_{rand}-r_{1}X_{rand}-2r_{2}X_{t},q\geq 0.5\\ \left(X_{\mathrm{rabbit},t}-X_{m,t}\right)-r_{3}\left(lb+r_{4}\left(ub-lb\right)\right),q<0.5 \end{array}\right.$$

In the equation, $X_{t}$ and $X_{t+1}$ are the position vector of the Harris Hawk at the iterations *t* and *t* + 1, respectively, $X_{\mathrm{rabbit},t}$ is the position of the rabbit (prey) after the *t* iteration, and $X_{rand,t}$ is the randomly selected hawk from the current population at iteration *t*. $X_{m,t}$ is the average position of the Harris hawk in iteration *t*, *lb* and *ub* are the lower and upper bounds of the search space, respectively, *q* and $r_{1}$, $r_{2}$, $r_{3}$, and $r_{4}$ are random numbers in the interval (0, 1), which The equation is:

(6) $$X_{m,t}=\frac{1}{N}\sum_{i=1}^{n}X_{i,t}$$

#### 3.2.2. Transition from Exploration to Exploitation

The operation of the swarm optimization algorithm needs to maintain a balance between exploration to exploitation. The Harris hawk can switch between different exploitative behaviors based on the escaping energy of the prey. During the escape behavior of the prey, its escape energy *E* will be greatly reduced. HHO also uses the escape energy equation to complete the conversion between mining and exploration, and its expression is as follows:

(7) $$E=2E_{0}\left(1-\frac{t}{T}\right)$$

Among them, E indicates the escaping energy of the prey. $E_{0}$ denotes the initial state of its energy, and its expression is $E_{0}$ = 2*rand* − 1, and rand is a random number in [0, 1]. *t* denotes the current iteration round, and *T* denotes the maximum number of iterations. When |E|≥1, the Harris Hawks Optimization is in the exploration stage, otherwise it is in the local mining stage.

#### 3.2.3. Exploitation Phase

In this phase, the exploitation phase is accomplished using four approaches at parameter sets. These approaches are based on the position identified in the exploration phase. Unfortunately, prey often escapes ahead of the Harris Hawk. Therefore, according to the escape behavior of the prey and its pursuit strategy, Harris Hawk has evolved four attack strategies, and the HHO algorithm also uses four strategies to simulate the four attacks of Harris Hawk. In this paper, *r* is used to denote the probability that the prey escapes successfully. When *r* < 0.5, the prey escapes successfully; When r≥0.5, the prey fails to escape, use the previous E to accompany you with Harris Hawk’s offensive strategy, when |E|≥0.5, adopt a soft siege, and when |E| < 0.5, execute a forced siege.

- Soft Besiege
  When r≥0.5 and |E|≥0.5, the prey has enough energy to escape, so the Harris Hawk uses a soft siege strategy, the main purpose of which is to consume the energy of the prey, and choose the best position to raid and dive to catch the prey, The equation for its position update is as follows:
  (8) $$X_{t+1}=\Delta X_{t}-E\left|JX_{\mathrm{rabbit},t}-X_{t}\right|$$
  (9) $$\Delta X_{t}=X_{\mathrm{rabbit},t}-X_{t}$$
  In the equation, $X_{\mathrm{rabbit},t}$ is the position of the prey at iteration *t*, $X_{t}$ is the difference between the position vector of the rabbit and the current location in iteration *t*, *J* = 2(1 − rand), which is the random jump strength of the rabbit throughout the escaping procedure, where rand is the a random number inside (0, 1).
- Hard Besiege
  When r≥0.5 and |E| < 0.5, the energy of the prey is severely consumed and exhausted. In addition, the Harris hawks hardly encircle the intended prey to finally perform the surprise pounce. The mathematical expression for its position update is:
  (10) $$X_{t+1}=X_{\mathrm{rabbit},t}-E\left|\Delta X_{t]}\right|$$
  (11) $$\Delta X_{t}=X_{\mathrm{rabbit},t}-X_{t}$$
- Soft besiege with progressive rapid dives
  When *r* < 0.5 and |E| > 0.5, the prey still has a chance to escape, and the escape energy is sufficient. The Harris Hawks would make a soft siege before attacking. In order to simulate the escape mode of the prey, HHO introduces the Levy function(LF) to update the mathematical expression of the position in the HHO algorithm:
  (12) $$X_{t+1}=\left\{\begin{array}{c} Y:X_{\mathrm{rabbit},t}-E\left|JX_{\mathrm{rabbit},t}-X_{t}\right|,if\;fitness\left(Y\right)<fitness\left(X_{t}\right)\\ Z:Y+S\times LF\left(D\right),if\;fitness\left(Z\right)<fitness\left(X_{t}\right) \end{array}\right.$$
  where *D* is the space dimension, and *S* is a 1 × *D* random vector, that is, *S* = rand(1,D); LF(D) is the Levy function:
  (13) $$LF=0.01\times \frac{\mu \times \delta }{{\left|\nu \right|}^{\frac{1}{\beta }}},\delta ={\left(\frac{\Gamma \left(1+\beta \right)\times \sin\left(\frac{\pi \times \beta }{2}\right)}{\Gamma \left(\frac{1+\beta }{2}\right)\times \beta \times 2^{\frac{\beta -1}{2}}}\right)}^{\frac{1}{\beta }}$$
  where *u* and *v* are random numbers uniformly distributed in [0,1], β=1.5.
- Hard besiege with progressive rapid dives
  When *r* < 0.5 and |E| < 0.5, the prey has a chance to escape, but the escape energy *E* is insufficient, so the Harris hawk adopts a hard besiege with progressive rapid dives, forming a hard besiege before the raid, and then shrinks them and the prey average distance. The mathematical expression for its position update is:
  (14) $$X_{t+1}=\left\{\begin{array}{c} Y:X_{\mathrm{rabbit},t}-E\left|JX_{\mathrm{rabbit},t}-X_{m,t}\right|,if\;fitness\left(Y\right)<fitness\left(X_{t}\right)\\ Z:Y+S\times LF\left(D\right),if\;fitness\left(Z\right)<fitness\left(X_{t}\right) \end{array}\right.$$
  The HHO uses escape energy *E* and factor *r* to configure four attack mechanisms between Harris hawk and prey to solve the optimization problem.

## 4. Elevated HHO

### 4.1. Elevated HHO for Escape Energy Function

The structure of the traditional Harris Hawks Optimization itself has certain defects, and the search process is prone to the problems of falling into local optimum and low convergence accuracy. The escape energy equation E is used to adjust the exploration and exploitation phases and four attack strategies. The higher the value of E, the more HHO tends to perform exploration, and vice versa for exploitation. However, in traditional HHO, E decreases linearly with the number of iterations, which will cause the algorithm to be biased towards local search and easily fall into local optimum. Therefore, an improved energy escape function E is proposed in this paper, and a special exponential function is introduced to adjust the exploration and exploitation phases, so it makes the optimization algorithm more inclined to perform a global search. Its corresponding energy equation is:

(15) $$E=2\times E_{0}\times \left(2\times rand\times e^{\omega }\right),\omega =-\frac{\pi t}{2T}$$

where rand is a random number inside (0, 1), *t* denotes the current iteration round, and T denotes the maximum number of iterations.

### 4.2. Elevated HHO for Random Jump Distance Function

The prey jumping distance *J* in the HHO cannot well simulate the trend of the prey changing with the decay of the energy for the position update equation. Therefore, this paper proposes a jumping distance equation *J* that changes with the escape energy to better simulate the position of the prey, and make the optimization algorithm better explore the space of different regions. The improved jump distance equation is:

(16) $$J=4\times \left(2\times rand\times e^{-\frac{\pi t}{2T}}\right)\times \left(1-rand\right)$$

where rand is a random number inside (0, 1), *t* denotes the current iteration round, and *T* denotes the maximum number of iterations. This elevated HHO algorithm will be referred to as EHHO hereinafter. The pseudo code of the HHO algorithm is shown in Algorithm 1, and the pseudo code of EHHO is shown in Algorithm 2.

**Algorithm 1:** Pseudo code of standard Harris Hawks Optimisation.
**Input**: The population size N and maximum number of iterations T
**Output**: The location of rabbit and its fitness value
Initialize the random population $X_{i}\left(i=1,2,...,N\right)$


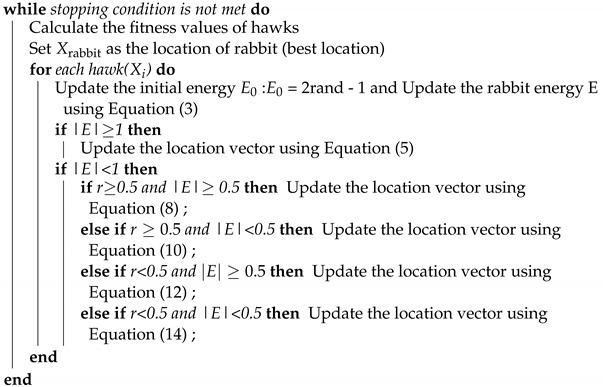



**return** $X_{\mathrm{rabbit}}$




**Algorithm 2:** Pseudo code of Elevated Harris Hawks Optimisation.
**Input**: The population size N and maximum number of iterations T
**Output**: The location of rabbit and its fitness value
Initialize the random population $X_{i}\left(i=1,2,...,N\right)$


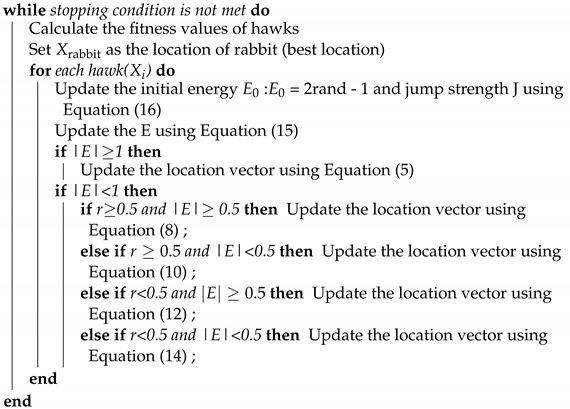



**return** $X_{\mathrm{rabbit}}$




### 4.3. Fitness Function

The selection of the fitness function directly affects the convergence speed of the optimization algorithm and whether it can find the optimal solution, because the optimization algorithm basically does not use external information in the process of optimization, and only uses the fitness function as the basis to use the adaptation of each individual in the population degree to search. Because the complexity of the fitness function is the main component of the complexity of the entire anomalous network traffic detection method, the design of the fitness function should be as simple as possible to minimize the time complexity of the calculation. Accordingly, this paper designs the fitness function of the HHO as follows:

(17) $$C=\alpha \times \left(1-acc\right)+\beta \times \frac{F}{F_{max}}$$

Among them, α is the accuracy weight constant, set to 0.99, acc is the classification accuracy using the DT classifier, and β is the step weight constant, set to 0.01. where F is the feature subset, and $F_{max}$ is the total feature count. It can be seen from Equation (17) that the higher the classification accuracy and the lower the dimension of the feature subset, the better the fitness value of the EHHO algorithm.

### 4.4. Detailed Execution Flow of EHHO-GRU Abnormal Flow Detection Method

1. Data preprocessing. Delete the feature columns with more than half of the total missing values in the malware detection dataset, map discrete data into feature columns with one-hot encoding, and process the missing values in the dataset with 0 padding; in binary classification, set the class label to normal and anomalies are coded 0 and 1 respectively; the data set is processed in a normalized way to reduce the influence of the data dimension, and the features that are completely irrelevant to the label column in the data set are deleted in the way of mutual information feature selection.
2. Optimization algorithm initialization. Set the initialization parameters of the EHHO algorithm population, the number of iterations, and the problem dimension, and determine the fitness function. Initialize the population and perform binary discretization of the feature dimensions in feasible solutions.
3. Calculate the fitness. Taking the fitness function as the optimization direction, calculating the fitness value of the feasible solution after iteration, taking the feasible solution with the best fitness in the population as the prey, and record the optimal individual information.
4. Determine whether the optimization cycle is terminated, if the termination condition is met, go to step (5); otherwise, go back to step (3).
5. Output the optimal feature subset and send it to the GRU classifier, and end the algorithm process.

**Figure 2:**
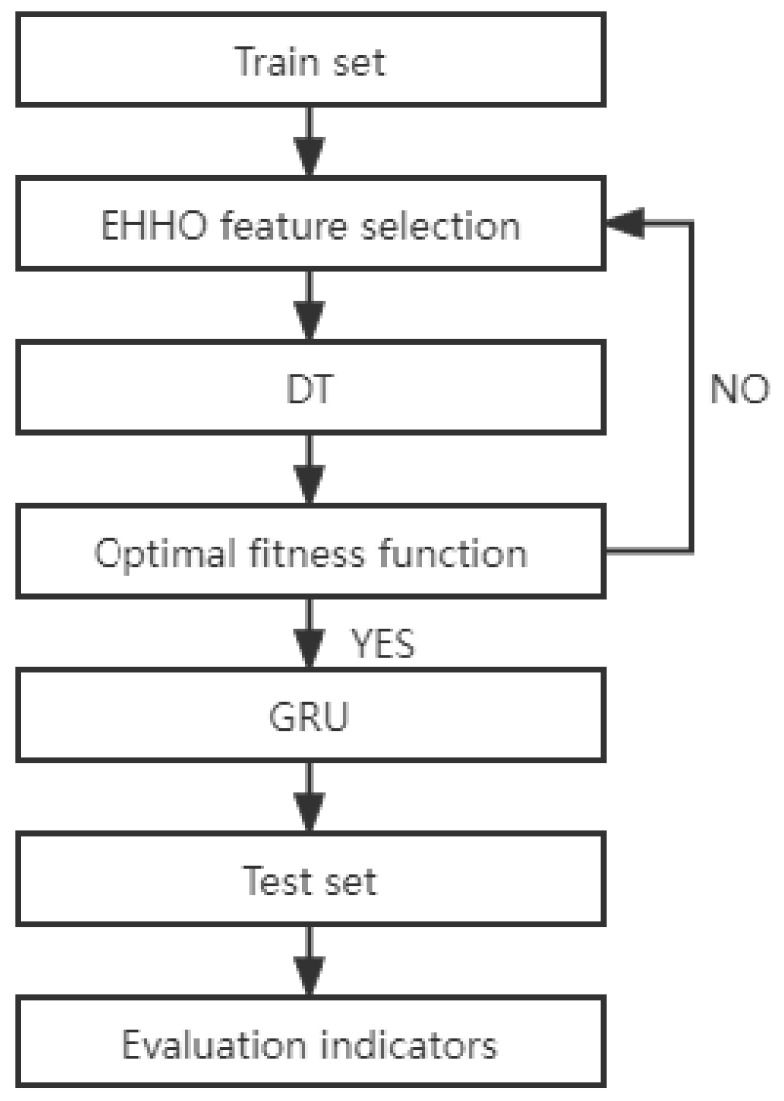
The overall process.

The overall process is shown in Figure 2.

## 5. Experiment

### 5.1. Experiment Environment

In this paper, the specific experimental environment is shown in Table 1. I chose PyTorch between PyTorch and Keras framework because it is more suitable for academic research.

### 5.2. Use of Datasets

In this paper, NSL-KDD [17], UNSW-NB15 [18] and CICIDS2018 datasets were used to test the EHHO-GRU anomaly detection method. The NSL-KDD dataset and UNSW-NB15 dataset both have divided training and test sets, while the CICIDS dataset is selected to be subdivided into training and test sets for 10% of the data due to the limitation of the experimental environment.

- NSL-KDD dataset
  The NSL-KDD dataset is a simplified and improved version of the KDDCUP99 dataset. The NSL-KDD dataset ensures that the intrusion detection model is free from bias and is more suitable for deep learning for anomalous traffic monitoring. The classification labels in the multiclassification test are shown in Table 2, which gives details of the NSL-KDD dataset. In the dichotomous test the classification labels are all 1 except for the Normal label which is 0. The detailed composition is shown in Table 2.

**Table 2:** Composition of NSL-KDD dataset.

Attack Category	Description	Train	Test
Normal	normal flow record	67,341	9711
Probe	Get detailed statistics on system and network configuration	11,656	7456
DoS	Attacks are designed to degrade network resources	45,927	2421
U2R	get permission	114	1436
R2L	Illegal access to a remote computer	934	1520
Total		125,972	22,543

- UNSW-NB15 dataset
  The UNSW-NB15 dataset is a new public dataset introduced by the Cyber Security Experimentation Team at the Australian Cyber Security Centre. Cyber security researchers often use the UNSW-NB15 dataset to address issues identified in the NSL-KDD dataset and the KDDCUP99 dataset. The dataset is generated in a hybrid manner and includes both normal and attack traffic from real-time network traffic, making it a comprehensive dataset of network attack traffic. There was a total of 49 features in the dataset to describe a piece of data. There are a total of nine types of abnormal attack traffic marked as 1 and one type of normal traffic marked as 0 in this dataset, the details of which are shown in Table 3 below.

**Table 3:** Composition of UNSW-NB15 dataset.

Attack Category	Description	Train	Test
Normal	normal flow record	37,000	56,000
Backdoor	Techniques to gain access to programs or systems by bypassing security controls	583	1746
Analysis	Intrusion methods of infiltrating web applications through ports and web scripts	677	2000
Fuzzers	An attack that tries to find a security hole by passing a lot of random data, making it crash	6062	18,184
Shellcode	Attacks that control the target machine by sending code that exploits a specific vulnerability	378	1133
Reconnaissance	Attacks that collect computer network information to evade security controls	3496	10,491
Exploit	Code that takes control of the target system by triggering a bug or several bugs	11,132	33,393
DoS	Attacks are designed to degrade network resources	4089	12,264
Worms	Actively attacking malignant computer virus spread through the network	44	130
Genertic	A technique for colliding each block cipher using a hash function	18,871	40,000
Total		82,332	175,341

- CICIDS2018 dataset
  The CICIDS2018 dataset is a collaborative project between the Communications Security Establishment (CSE) and the Canadian Institute for Cybersecurity Research (CIC). Previous partial datasets were highly anonymous, did not reflect current trends, or they lacked certain statistical characteristics for which perfect datasets existed. The Canadian research team has therefore devised a systematic approach to generating datasets to analyze, test, and evaluate intrusion detection systems, with a focus on network-based anomaly detectors. The dataset includes seven different attack scenarios: Brute-force, Heartbleed, Botnet, DoS, DDoS, Web attacks, and more. The data is made up of an attack machine consisting of 50 hosts, with 5 sectors of target machines, including 420 machines and 30 servers. The dataset includes network traffic and system logs for each machine captured, as well as 80 features extracted from the captured traffic using CICFlowMeter-V3. As the dataset is too large to contain ten datasets and is limited by the experimental environment, a portion of the data from each dataset is selected to form a new dataset for the experiment, and 10% of each dataset is extracted hierarchically for generalizability. There are seven types of abnormal attack traffic marked as 1 and one type of normal traffic marked as 0 in this dataset, and the detailed composition is shown in Table 4.

### 5.3. Dataset Preprocessing Step

- Data normalisation
  A min-max normalization process is used to compress the data into the (−1, 1) interval. One advantage is that it improves the speed of convergence of the model, and another is that it improves the accuracy of convergence, which is given by:
  (18) $$X^{\prime }=\frac{X-X_{min}}{X_{max}-X_{min}}$$
  where $X_{min}$ denotes the minimum value that occurs after normalization of all samples for that dimensional feature, $X_{max}$ denotes the maximum value that occurs after normalization of all samples for that dimensional feature, and *x*${}^{\prime }$ denotes the result of normalization of each data sample.
- Character feature unique heat code
  In feature engineering, data will appear as category-based features, including character-based features and discontinuous features. In order to solve the problem that the classifier does not handle attribute data well, this paper uses the pre-processing part of the Keras framework to process these category-based data with unique thermal coding.
- Dataset labels
  Some datasets commonly used in the field of anomalous traffic detection will have more than one label, due to the characteristics of the data commonly used in this field, which, in addition to labeling the different attack method category labels, also typically use 0: for normal network traffic, 1: for abnormal network traffic, to act as labels for the network data. Some datasets (e.g., UNSW-NB15 dataset) inherently have two label columns, while in others (e.g., NSL-KDD dataset), only the attack category label column exists. In order to better evaluate the generalisability of the anomalous traffic detection model proposed in this paper, this paper tests and evaluates each dataset separately for multiple and dual classifications.

### 5.4. Experimental Model Parameter Settings

The hyperparameters of the anomalous traffic detection model proposed in this paper consist of two main parts, one is the hyperparameters of the EHHO feature selection part for iteration, and the other is the hyperparameters part of the two-layer GRU neural network classifier. The detailed parameters are shown in Table 5.

In the feature selection part of this paper, four novel nature-inspired algorithms with good optimization search results - the Whale Optimisation Algorithm [19], the Genetic Algorithm [20], the Particle Swarm Optimisation Algorithm [21], and the basic Harris Hawks Optimisation(HHO) are selected for comparison with the elevated Harris Hawk Optimisation(EHHO). To reflect the fairness and objectivity of the experiments, the population size N was set to 30 for all algorithms, the number of iterations T was set, and the common parameters of the four algorithms were kept the same.

The performance of the neural network classification model is influenced by the parameters selected.

### 5.5. Experimental Evaluation Metrics

The model evaluation criteria [22] are defined as follows:

TP (True Positive): True positives, which are actually intrusion samples and the number of successfully detected as intrusion samples; FN (False Negative): False negatives, which are actually intrusion samples but have not been correctly detected, that is, false negatives Number of samples; FP (False Positive): false positives, which are actually normal samples but detected as intrusion samples, that is, the number of false positive samples; TN (True Negative): true negatives, which are actually normal samples and are not falsely reported the number of samples.

Accuracy: This metric counts the ratio of the number of correctly classified samples to the entire test set. The higher the accuracy, the better the performance of the neural network model (Accuracy∈[0,1]). It is the most commonly used model evaluation index in the field of deep learning, and the accuracy rate is defined as:

(19) $$Accuracy=\frac{TP+TN}{TP+TN+FP+FN}$$

Precision: This indicator counts the ratio of correctly identified normal samples to the total number of predicted normal samples. The higher the precision, the better the performance of the neural network model (Precision∈[0,1]), and the precision rate is defined as:

(20) $$Precision=\frac{TP}{TP+FP}$$

Recall: This indicator counts how many positive examples in the sample are predicted correctly. The higher the precision, the better the performance of the neural network model (Recall∈[0,1]), and the recall is defined as:

(21) $$Recall=\frac{TP}{TP+FN}$$

F1-score: F1-Score is also known as F1-Measure. This metric counts the harmonic mean of precision and recall. The higher the F1-Score, the better the neural network model (F1−Score∈[0,1]). F1-Score is defined as follows:

(22) $$F1-score=\frac{2\times Precision\times Recall}{Precision+Recall}$$

True Positive Rate (TPR): This metric is also known as Recall, and its equation is the same as Recall. False Positive Rate (FPR): This metric counts the ratio of the number of attack samples predicted to be normal samples to the actual total number of attacks. The lower the FPR, the better the performance of the neural network model (FPR∈[0,1]). The definition of FPR is as follows:

(23) $$FPR=\frac{FP}{TN+FP}$$

## 6. Experimental Results and Analysis

### 6.1. EHHO Algorithm Performance Test

**Figure 3:**
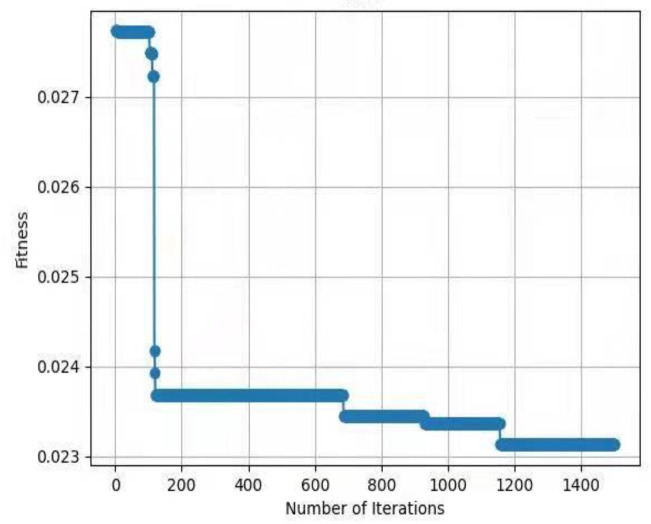
The fitness of HHO.

**Figure 4:**
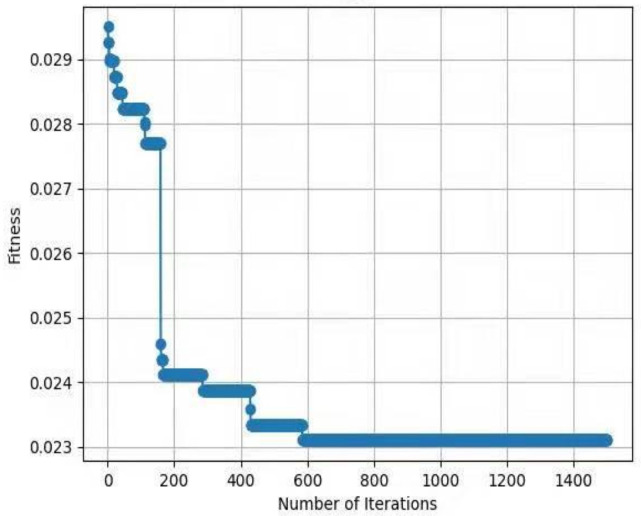
The fitness of EHHO.

For testing the performance of EHHO’s search, this paper uses HHO and EHHO, CICDIS2018 dataset to solve the feature subset respectively, with the maximum number of iteration rounds both set to 1500 and Max depth set to 4. Then the fitness functions of the two algorithms vary with the number of iteration rounds as shown in Figure 3 and Figure 4. From the two figures, we can see that HHO converges at close to 1200 rounds, while EHHO has converged at less than 600 rounds. The lower the fitness function, the better the selected feature subset, and the higher the fitness function when HHO converges than EHHO, which shows that the elevated EHHO outperforms HHO in terms of both convergence speed and convergence accuracy.

To better test the generality of the performance of the EHHO algorithm, we choose four test functions to evaluate the optimized performance of the EHHO algorithm. The four test functions are shown in Table 6.

**Figure 5:**
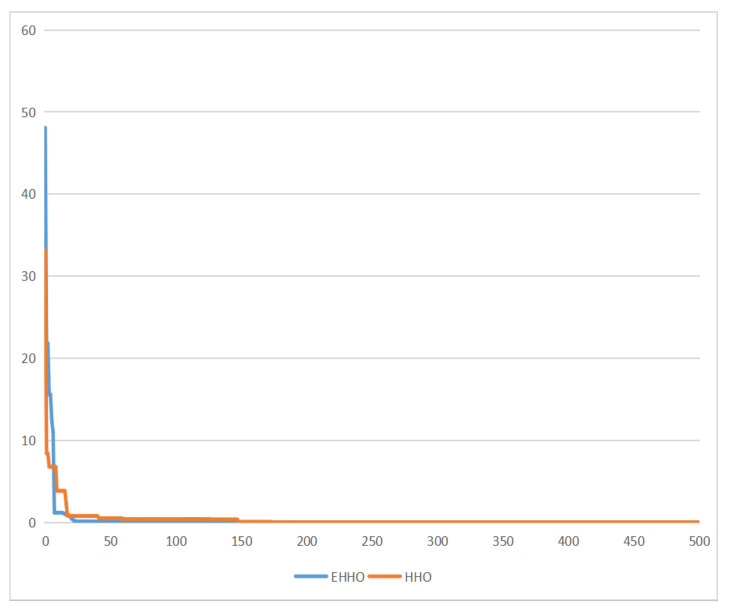
Rastrigin function.

**Figure 6:**
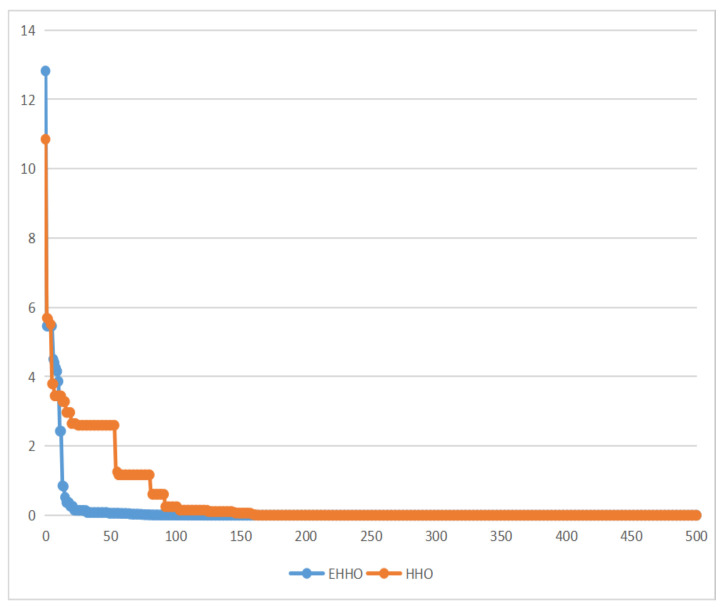
Ackley function.

**Figure 7:**
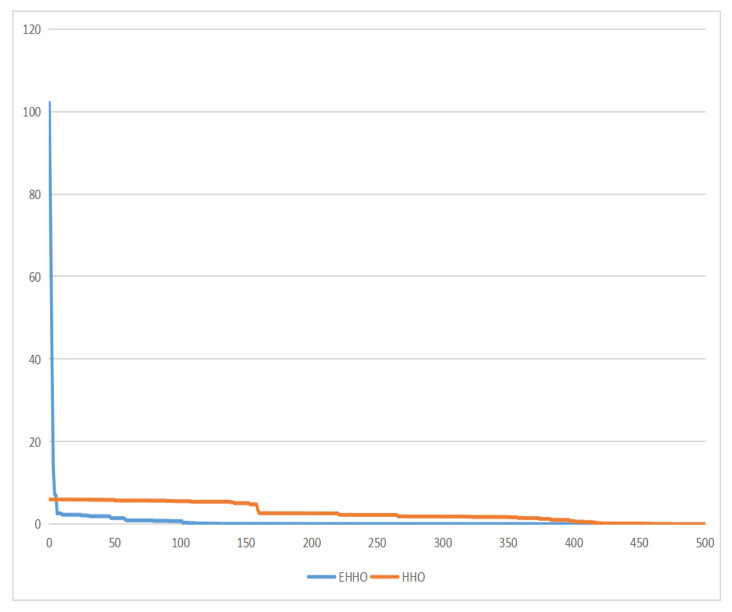
Booth function.

**Figure 8:**
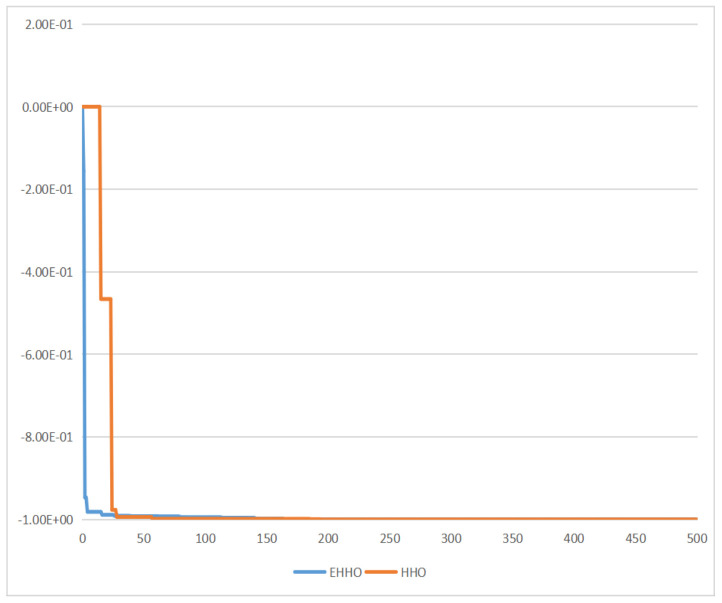
Easom function.

As shown in Figure 5, Figure 6, Figure 7 and Figure 8. EHHO is almost able to converge faster than HHO in the tests of all four functions. This can fully demonstrate that EHHO is better than HHO in both convergence accuracy and speed.

### 6.2. Analysis of the EHHO-GRU Model Results for the NSL-KDD Dataset

In the NSL-KDD dataset, it is important to note that 24 attack types appear in the training set and 38 attack types appear in the test set, meaning that 14 new attack types appear in the test set (and none in the training set). In order to address this situation which would lead to the neural network not being able to correctly classify the test set after training, this paper will first perform a binary classification test on the NSL-KDD dataset by setting the new label to 0 except for the one with the classification label of normal, and setting the abnormal traffic of the rest of the labels to 1. After this treatment, the test set contains labeled data that did not appear in the training set, which can better detect the detection model’s Generalisability. The number of iterations of the feature subset solving algorithm was 1500 and the number of iterations of the GRU neural network was 3000, and the detailed experimental results of the binary classification are shown in Table 7 below.

**Figure 9:**
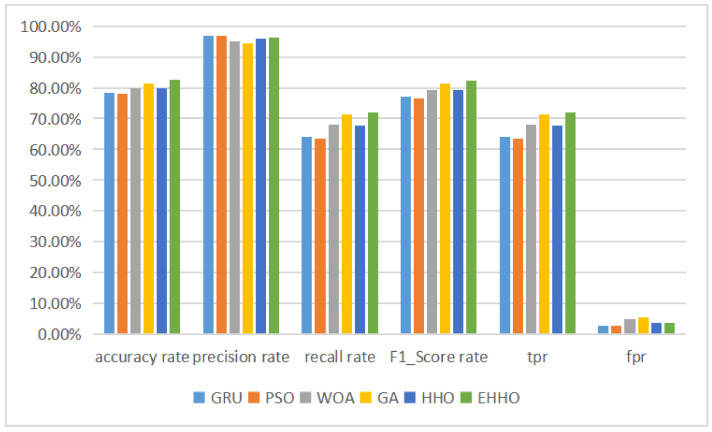
Binary classification histogram of NSL-KDD.

The histogram is shown in Figure 9.

According to the table above, it can be seen that after the neural network has completed training on the training set, the test set contains 14 labeled data that are not available in the training set, resulting in a GRU neural network without feature selection The accuracy of the detection model with the subset of features solved by the optimization algorithm and then fed into the neural network for training was higher than that of the neural network without feature selection, except for the Particle Swarm Optimisation (PSO) + GRU model, which was less accurate than the original version. The accuracy of the neural network classification was still higher than that of the model without feature selection, even though the feature dimensionality was sharply reduced to one-eighth of the original version. The EHHO+GRU model with the highest accuracy was 2.5% more accurate than the HHO+GRU model, and 4.13% more accurate than the model without feature selection, while sharply reducing 35 redundant features and having a higher recall and f1-score than the GRU model. And the multi-classification tests for the NSL-KDD dataset are shown in Table 8 below.

**Figure 10:**
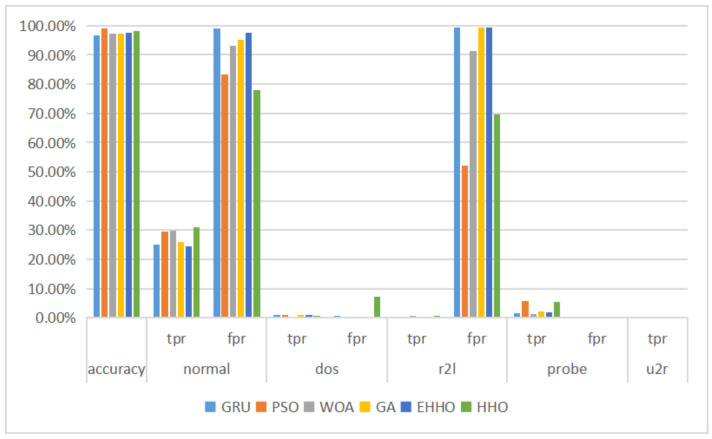
Multi-classification histogram of NSL-KDD.

As can be seen in Table 8 and Figure 10, the EHHO+GRU model is the most accurate of all, with only about one-eighth of the original dimensionality of the features selected.

### 6.3. Analysis of the EHHO-GRU Model Results for the UNSW-NB15 Dataset

**Figure 11:**
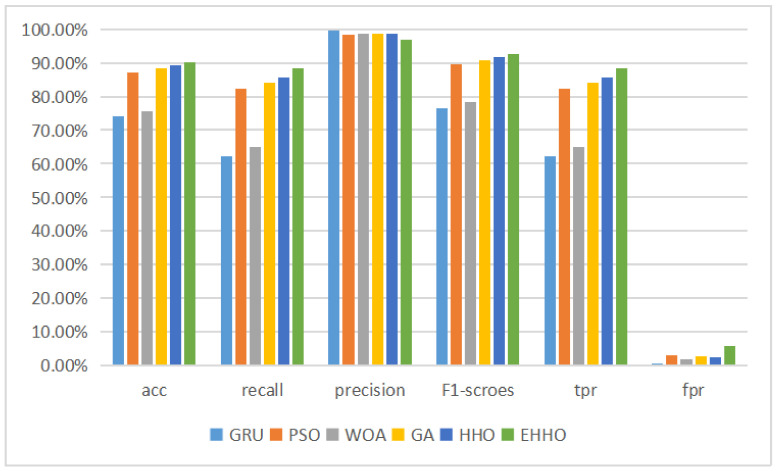
Binary classification histogram of UNSW-NB15.

After eliminating some redundant data columns such as dates, the UNSW-NB15 dataset is described in this paper using 42 features, which are labeled as 0 and nine different 1 types. Therefore both binary and multiclassification performance tests are conducted on this dataset in this paper, where the results of the binary classification experiments are shown in Table 9 and Figure 11 below.

This is because the training data in the UNSW-NB15 dataset is less than one-half of the test data, which reduces the classification performance of the normal GRU neural network on the test set. The remaining five detection models using the optimization algorithm for feature selection showed substantial improvements in all four evaluation metrics, with the HHO+GRU model achieving 89.33% classification accuracy using a subset of 11-dimensional features, an improvement of 15.25%, and 15.04% improvement in f1-score, while the elevated EHHO+GRU model achieved the highest accuracy despite using 20-dimensional features, but achieved the highest accuracy, recall and f1-score.

**Figure 12:**
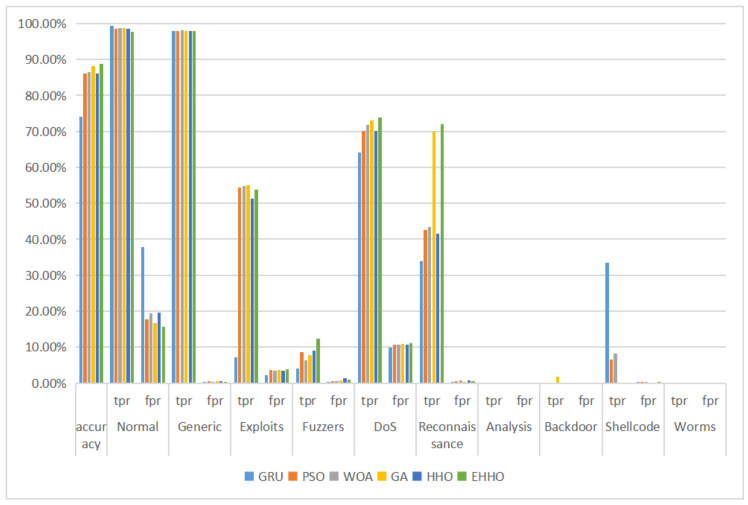
Multi-classification histogram of UNSW-NB15.

The paper next conducts a multi-category experiment on this dataset, the results of which are shown in the table below, where the data imbalance problem is particularly severe due to the under-representation of Analysis, Backdoor, Shellcode, and Worms. The statistical tables are shown in Table 10 and Figure 12.

As can be seen from the above table, the overall accuracy of the model using all 42-dimensional features is only the lowest at 74.01%, with a false positive rate of 37.86% for normal tags, which is particularly serious as an abnormal traffic detection model, meaning that a large amount of abnormal traffic is labeled as normal traffic. The overall accuracy of the detection models with optimized algorithm feature selection exceeds 85%, with the elevated EHHO+GRU model using a 22-dimensional feature subset having the highest overall accuracy, as well as the lowest false alarm rate for the normal tag and the highest tpr for the Fuzzers, DoS, and Reconnaissance tags, this shows that the proposed EHHO+GRU anomaly traffic monitoring model has the best overall performance in the UNSW-NB15 dataset for both binary and multi-classification performance evaluation.

### 6.4. EHHO-GRU Model Analysis of CICIDS2018 Dataset Results

**Figure 13:**
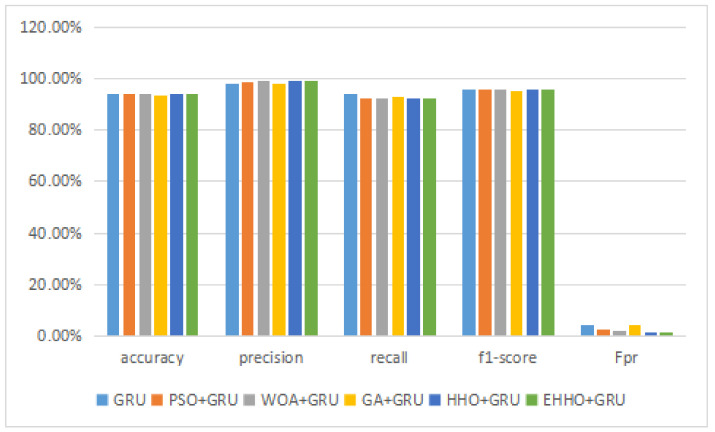
Binary classification histogram of CICIDS2018.

**Figure 14:**
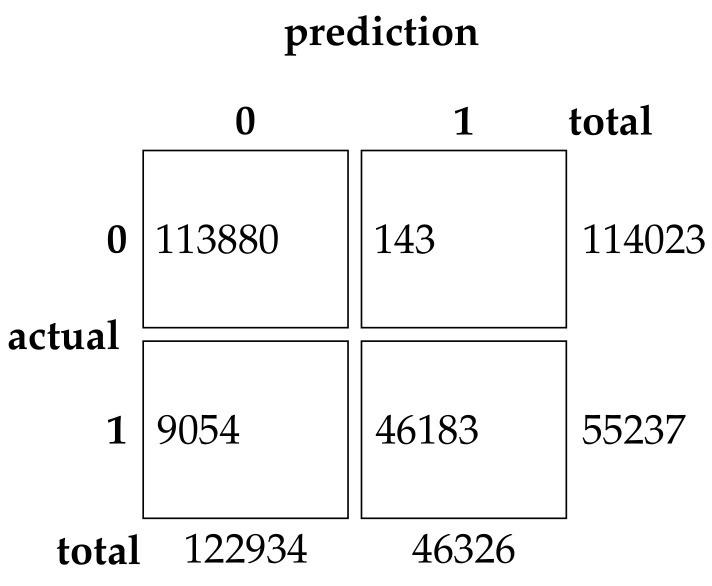
Confusion Matrix of GRU.

**Figure 15:**
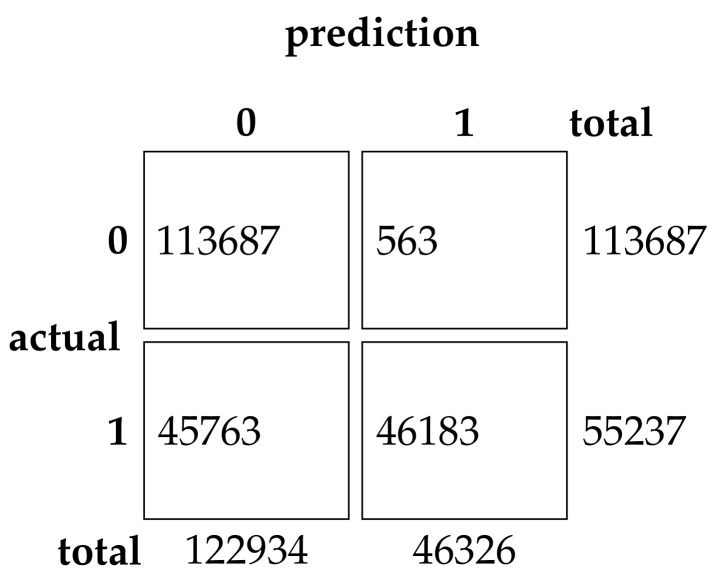
Confusion Matrix of EHHO.

This experimental dataset was experimented with batch processing due to the large amount of data, Batch size = 64, the number of iterations of the optimization algorithm $T_{0}=200$, and the number of iterations of the neural network was 200. The results of the binary classification experiment are shown in Table 11 and Figure 13.The confusion matrix of the classification results of the GRU method and the EHHO method in this dataset is shown in Figure 14 and Figure 15.

As can be seen from the table, the GRU model using all 80-dimensional features has the highest accuracy, but also has the highest false alarm rate. The training of data with 80-dimensional features is very slow and requires a lot of computational resources, while the detection model after feature selection can reduce the feature dimensionality to as low as one in sixteen, with a slight decrease in accuracy. In contrast, the EHHO+GRU model in this paper reduces the feature dimensionality to one-eighth, with a 0.37% decrease in accuracy, and fpr also only increased by 0.9%.

## 7. Conclusions

The Harris Hawks optimization algorithm has been a relatively new meta-heuristic optimization algorithm in recent years. The experimental results of binary and multi-classification of NSL-KDD and UNSW-NB15 datasets show that the model has better performance, which not only greatly reduces the feature dimension of the dataset, but also reduces the learning time of the neural network, and can also improve the accuracy, precision, recall, and f1-score of the anomalous network traffic detection system. The EHHO-GRU method is used to select features and dimensionality reduction even reaches 90% in the CICIDS2018 data set. However, the method is not tested on the full CICIDS2018 dataset owing to its size and can’t address the data imbalance problem that occurred in the UNSW-NB15 dataset, causing a very low accuracy in classifying a relatively small proportion of labels. Since the study focuses on metrics such as the accuracy of the detection method on the test set, EHHO’s testing is relatively simple. From there, in the future work I will focus on improving the generality of the proposal and testing it in a real experimental environment. 

## Figures and Tables

**Table 1:** Experimental environment.

CPU	AMD R5 3600X
GPU	Nvdia rtx2060
RAM	16 GB
Language	Python3.9
Deep Learning Framework	Pytorch

**Table 4:** Composition of CICIDS2018 dataset.

Attack Category	Description	Train	Test
Brute-force attack	Perform brute force and password cracking attacks	31,767	21,178
Botnet	botnet	17,167	11,445
DoS	Attacks are designed to degrade network resources	38,606	25,738
DDoS	Distributed Denial of Service Attack	82,307	54,871
Infiltration	Intranet penetration attack	13,640	9093
SQL	SQL injection attack	8	5
Benign	benign traffic	69,489	46,326
Total		252,984	168,656

**Table 5:** Detailed experimental hyperparameters.

Category	Description	
N	initial population	30
T${}_{O}$	The maximum number of iterations of the feature selection algorithm	See the specific experiment section for details.
Max depth	Decision tree maximum depth	4
Hid dim	The number of hidden layer units in the neural network	128
Lr	Neural network learning rate	0.0005
E	The number of neural network iterations	3000
φ	Neural network forgetting rate	0.5

**Table 6:** Test the function variable scale.

Function	Equation	Variable Domain	The Optimal Value
Ackley	$f\left(x\right)=-20*\;e^{-0.2*\;\sqrt{\frac{1}{n}\sum_{j=1}^{n}x_{j}^{2}}}-\;e^{\frac{1}{n}\sum_{j=1}^{n}\cos\left(2\pi x_{j}\right)}+\;22.71282$	[−5, 5]	0
Booth	$f\left(x\right)=\;{\left(x_{1}+2x_{2}-7\right)}^{2}+\;{\left(2x_{1}+x_{2}-5\right)}^{2}$	[−10, 10]	0
Easom	$f_{\mathrm{Easo}\;}\left(x_{1},x_{2}\right)=\;-\cos\left(x_{1}\right)\cdot \cos\left(x_{2}\right)\cdot \;e^{-\left({\left(x_{1}-\pi \right)}^{2}+{\left(x_{2}-\pi \right)}^{2}\right)}$	[−100, 100]	−1
Rastrigin	$\mathrm{Ras}\left(x\right)=\;20+x_{1}^{2}+x_{2}^{2}-\;10\left(\cos2\pi x_{1}+\cos2\pi x_{2}\right)$	[−5.12, 5.12]	0

**Table 7:** Experimental results of NSL-KDD binary classification.

Method	GRU	PSO	WOA	GA	HHO	EHHO
Feature dimension	41	11	5	5	5	6
accuracy	78.34%	77.94%	79.77%	81.42%	79.97%	82.47%
precision	96.84%	96.79%	95.02%	94.58%	96.07%	96.23%
recall	64.03%	63.34%	68.03%	71.46%	67.58%	72.02%
f1-score	77.09%	76.57%	79.29%	81.41%	79.34%	82.38%
Fpr	2.76%	2.78%	4.72%	5.41%	3.66%	3.73%

**Table 8:** Multi-classification experimental results of NSL-KDD.

Method	GRU	PSO	WOA	GA	HHO	EHHO
Feature dimension	42	9	8	10	9	8
accuracy	86.27%	85.14%	84.14%	86.13%	84.13%	86.85%
normal tpr	96.72%	98.89%	97.11%	97.29%	98.23%	97.52%
normal fpr	24.89%	29.56%	29.71%	25.81%	30.95%	24.55%
DoS tpr	98.92%	83.23%	93.12%	95.21%	77.83%	97.46%
DoS fpr	0.43%	1.06%	0.48%	0.38%	0.54%	0.36%
r2l tpr	0.23%	0.00%	0.00%	0.00%	7.05%	0.00%
r2l fpr	0.04%	0.00%	0.07%	0.03%	0.28%	0.02%
probe tpr	99.37%	52.08%	91.32%	99.28%	69.62%	99.46%
probe fpr	1.67%	5.73%	1.32%	2.17%	5.27%	1.97%
u2r tpr	0.00%	0.00%	0.00%	0.00%	0.00%	0.00%
u2r fpr	0.00%	0.00%	0.00%	0.00%	0.00%	0.00%

**Table 9:** The results of the UNSW-NB15 binary classification experiment.

Method	GRU	PSO	WOA	GA	HHO	EHHO
Feature dimension	42	19	23	18	11	20
accuracy	74.08%	87.04%	75.56%	88.37%	89.33%	90.26%
precision	99.59%	98.31%	98.7%	98.57%	98.62%	96.96%
recall	62.18%	82.38%	64.94%	84.13%	85.51%	88.46%
f1-score	76.56%	89.64%	78.34%	90.78%	91.6%	92.52%
Fpr	0.55%	3.02%	1.82%	2.59%	2.54%	5.92%

**Table 10:** The results of the UNSW-NB15 multi-classification experiment.

Method	GRU	PSO	WOA	GA	HHO	EHHO
Feature dimension	42	19	11	16	16	22
accuracy	74.01%	85.97%	86.45%	88.17%	86.14%	88.67%
Normal TPR	99.3%	98.41%	98.79%	98.62%	98.45%	97.76%
Normal FPR	37.86%	17.77%	19.35%	16.74%	19.64%	15.6%
Generic TPR	97.87%	97.9%	98%	97.78%	97.85%	97.78%
Generic FPR	0.1%	0.21%	0.33%	0.15%	0.14%	0.04%
Exploits TPR	7.06%	54.3%	54.76%	54.95%	51.18%	53.66%
Exploits FPR	2.09%	3.74%	3.46%	3.62%	3.52%	3.89%
Fuzzers TPR	4.09%	8.66%	6.42%	7.8%	9.07%	12.4%
Fuzzers FPR	0.37%	0.59%	0.51%	0.66%	1.41%	0.99%
DoS TPR	64.2%	70.14%	71.71%	72.97%	70.21%	73.88%
DoS FPR	9.85%	10.6%	10.73%	10.96%	10.69%	11.02%
Reconnaissance TPR	33.92%	42.64%	43.35%	69.96%	41.46%	71.9%
Reconnaissance FPR	0.09%	0.44%	0.67%	0.41%	0.66%	0.6%
Analysis TPR	0%	0%	0%	0%	0%	0%
Analysis FPR	0%	0%	0%	0%	0%	0%
Backdoor TPR	0%	0%	0%	1.83%	0%	0%
Backdoor FPR	0%	0%	0%	0%	0%	0%
Shellcode TPR	33.54%	6.62%	8.21%	0%	0%	0%
Shellcode FPR	0.35%	0.09%	0.04%	0%	0%	0.01%
Worms TPR	0%	0%	0%	0%	0%	0%
Worms FPR	0%	0%	0%	0%	0%	0%

**Table 11:** The results of the CICIDS2018 binary classification experiment.

Method	GRU	PSO	WOA	GA	HHO	EHHO
Feature dimension	80	18	6	5	11	11
accuracy	94.57%	93.94%	94.06%	93.66%	94.15%	94.20%
precision	99.87%	98.93%	98.33%	98.3%	99.38%	99.51%
recall	92.64%	92.65%	93.41%	92.87%	93.41%	92.48%
f1-score	96.12%	95.69%	95.81%	95.51%	95.83%	95.86%
Fpr	0.31%	2.65%	4.22%	4.26%	1.53%	1.22%

## Data Availability

Not applicable.

## References

[1] Almomani O. (2020). A Feature Selection Model for Network Intrusion Detection System Based on PSO, GWO, FFA and GA Algorithms. Symmetry.

[2] Gu J., Wang L., Wang H., Wang S. (2019). A novel approach to intrusion detection using SVM ensemble with feature augmentation. Comput. Secur..

[3] Song K., Yan F., Ding T., Gao L., Lu S. (2020). A steel property optimization model based on the XGBoost algorithm and improved PSO. Comput. Mater. Sci..

[4] Júnior D.A.D., da Cruz L.B., Diniz J.O.B., da Silva G.L.F., Junior G.B., Silva A.C., de Paiva A.C., Nunes R.A., Gattass M. (2021). Automatic method for classifying COVID-19 patients based on chest X-ray images, using deep features and PSO-optimized XGBoost. Expert Syst. Appl..

[5] Aghdam M.H., Kabiri P. (2016). Feature selection for intrusion detection system using ant colony optimization. Int. J. Netw. Secur..

[6] Mazini M., Shirazi B., Mahdavi I. (2019). Anomaly network-based intrusion detection system using a reliable hybrid artificial bee colony and AdaBoost algorithms. J. King Saud Univ.-Comput. Inf. Sci..

[7] Zhang Y., Li P., Wang X. (2019). Intrusion detection for IoT based on improved genetic algorithm and deep belief network. IEEE Access.

[8] Zhang L., Fan X., Xu C. A fusion financial prediction strategy based on RNN and representative pattern discovery. Proceedings of the 2017 18th International Conference on Parallel and Distributed Computing, Applications and Technologies (PDCAT).

[9] Sheikhan M., Jadidi Z., Farrokhi A. (2012). Intrusion detection using reduced-size RNN based on feature grouping. Neural Comput. Appl..

[10] Agarap A.F.M. A neural network architecture combining gated recurrent unit (GRU) and support vector machine (SVM) for intrusion detection in network traffic data. Proceedings of the 2018 10th International Conference on Machine Learning and Computing.

[11] Zhang H., Kang C., Xiao Y. (2021). Research on Network Security Situation Awareness Based on the LSTM-DT Model. Sensors.

[12] Sak H., Senior A., Beaufays F. (2014). Long short-term memory based recurrent neural network architectures for large vocabulary speech recognition. arXiv.

[13] Li Y., Lu Y. LSTM-BA: DDoS detection approach combining LSTM and Bayes. Proceedings of the 2019 Seventh International Conference on Advanced Cloud and Big Data (CBD).

[14] Cho K., Van Merriënboer B., Gulcehre C., Bahdanau D., Bougares F., Schwenk H., Bengio Y. (2014). Learning phrase representations using RNN encoder-decoder for statistical machine translation. arXiv.

[15] Heidari A.A., Mirjalili S., Faris H., Aljarah I., Mafarja M., Chen H. (2019). Harris hawks optimization: Algorithm and applications. Future Gener. Comput. Syst..

[16] Chen H., Jiao S., Wang M., Heidari A.A., Zhao X. (2020). Parameters identification of photovoltaic cells and modules using diversification-enriched Harris hawks optimization with chaotic drifts. J. Clean. Prod..

[17] Protić D.D. (2018). Review of KDD Cup 99, NSL-KDD and Kyoto 2006+ datasets. Vojnoteh. Glas. Tech. Cour..

[18] Moustafa N., Slay J. (2016). The evaluation of Network Anomaly Detection Systems: Statistical analysis of the UNSW-NB15 data set and the comparison with the KDD99 data set. Inf. Secur. J. A Glob. Perspect..

[19] Mirjalili S., Lewis A. (2016). The whale optimization algorithm. Adv. Eng. Softw..

[20] Deb K., Pratap A., Agarwal S., Meyarivan T. (2002). A fast and elitist multiobjective genetic algorithm: NSGA-II. IEEE Trans. Evol. Comput..

[21] Poli R., Kennedy J., Blackwell T. (2007). Particle swarm optimization. Swarm Intell..

[22] Vinayakumar R., Alazab M., Soman K., Poornachandran P., Al-Nemrat A., Venkatraman S. (2019). Deep learning approach for intelligent intrusion detection system. IEEE Access.

